# Bone marrow adipocytes promote tumor growth in bone via FABP4-dependent mechanisms

**DOI:** 10.18632/oncotarget.1482

**Published:** 2013-10-29

**Authors:** Mackenzie K. Herroon, Erandi Rajagurubandara, Aimalie L. Hardaway, Katelyn Powell, Audrey Turchick, Daniel Feldmann, Izabela Podgorski

**Affiliations:** ^1^ Department of Pharmacology, Wayne State University School of Medicine, Detroit, MI; ^2^ Pathology, Wayne State University School of Medicine, Detroit, MI; ^3^ Karmanos Cancer Institute, Wayne State University School of Medicine, Detroit, MI; ^4^ Therapeutic Radiology, Yale School of Medicine

**Keywords:** bone marrow adipocytes, bone metastasis, prostate cancer, breast cancer, interleukin 1ß, heme oxygenase 1

## Abstract

Incidence of skeletal metastases and death from prostate cancer greatly increases with age and obesity, conditions which increase marrow adiposity. Bone marrow adipocytes are metabolically active components of bone metastatic niche that modulate the function of neighboring cells; yet the mechanisms of their involvement in tumor behavior in bone have not been explored. In this study, using experimental models of intraosseous tumor growth and diet-induced obesity, we demonstrate the promoting effects of marrow fat on growth and progression of skeletal prostate tumors. We reveal that exposure to lipids supplied by marrow adipocytes induces expression of lipid chaperone FABP4, pro-inflammatory interleukin IL-1β, and oxidative stress protein HMOX-1 in metastatic tumor cells and stimulates their growth and invasiveness. We show that FABP4 is highly overexpressed in prostate skeletal tumors from obese mice and in bone metastasis samples from prostate cancer patients. In addition, we provide results suggestive of bi-directional interaction between FABP4 and PPARγ pathways that may be driving aggressive tumor cell behavior in bone. Together, our data provide evidence for functional relationship between bone marrow adiposity and metastatic prostate cancers and unravel the FABP4/IL-1β axis as a potential therapeutic target for this presently incurable disease.

## INTRODUCTION

Prostate cancer preferentially metastasizes to skeletal sites and once established in the bone marrow becomes a devastating and incurable disease. Advanced age, obesity and associated metabolic pathologies are considered significant risk factors for development of skeletal metastases [1-5], but the mechanisms linking them to poor prostate cancer outcome are not well understood. One established feature of aging is a progressive infiltration of bone marrow by fat cells [6-8], a process that can be further exacerbated by obesity and related metabolic conditions [7, 9-14]. It has been estimated that in a 20-year old adult, approximately 15% of marrow space is filled with fat cells, a number that increases to 60% in a 65-year old person [7, 15, 16]. A conversion of hematopoietically active red marrow to fatty yellow marrow occurs first and is more predominant in appendicular as compared to axial skeleton [7, 8]; however by age 70 even the red marrow within the axial sites contains more than 50% fat [17, 18].

It has been previously demonstrated that prostate cancer cells are attracted to adipocytes within the metabolically active red bone marrow where metastases commonly occur [19, 20]. Previously considered to have little biological significance, bone marrow adipocytes are now recognized as metabolically active cells with specialized functions [8, 21]. They store and secrete fatty acids, cytokines, and adipokines, and have a potential to influence neighboring cells by autocrine, paracrine and endocrine mechanisms [8, 13, 22, 23]. Increased marrow adiposity due to diet-induced obesity in mice was recently implicated in altered bone metabolism and inflammation within the bone microenvironment [13, 14]. Bone marrow fat cells appear capable of translocating stored lipids to the metastatic tumor cells [20, 24], a process suggested to increase tumor cell motility [19]. The phenomenon of lipid-driven acceleration of growth and invasiveness was recently demonstrated in omental metastases from ovarian cancer [25], but has not been studied in a context of progression of metastatic tumors in bone. Epidemiological evidence shows that obese men have more than 3-fold higher risk of developing metastatic disease compared to normal weight men receiving the same treatment [5]; however, how adipocyte-rich bone marrow niche influences growth and survival of tumor cells that have metastasized to skeletal sites is currently not understood.

The key factor implicated in adipocyte-tumor cell interactions in ovarian cancer is a lipid chaperone, fatty acid binding protein 4 (FABP4) [25, 26]. FABP4 is predominantly known for its expression in adipocytes, macrophages and endothelial cells, where it is transcriptionally controlled by fatty acids, agonists of PPARγ (peroxisome proliferator associated receptor γ) and insulin [27, 28]. Although considered as a PPARγ target gene whose expression correlates with PPARγ activation [29], FABP4 also functions as PPARγ regulator by delivering its nuclear ligands and regulating signal transduction in a positive feedback loop [30]. Both PPARγ and FABP4 appear to be involved in adipocyte-induced metabolic switching in the prostate microenvironment that may be driving tumorigenesis [31]. Specifically, PPARγ signaling is reported to be suppressed by chronic administration of high fat diet [31], which disrupts metabolic oversight and results in malignant transformation [32]. Notably, the consequences of repressing PPARγ activity also include the initiation of inflammatory pathways, specifically the activation of NFκB (nuclear factor kappa B) [33] and downstream upregulation of IL-1β axis [34]. This further underlines the link between cellular metabolism and neoplastic progression.

The objective of the present study was to investigate the mechanisms behind the bone marrow adipocyte involvement in growth and progression of metastatic tumor cells in bone. Using combined models of diet-induced marrow adiposity and intratibial tumor growth we demonstrate that increased numbers of fat cells in the bone marrow due to high fat diet correlate with accelerated progression of skeletal tumors in mice. We show that lipid trafficking between marrow adipocytes and cancer cells fuels tumor growth and invasiveness by upregulating FABP4, as well as interleukin 1β (IL-1β), cytokine linked to tumor growth, invasiveness, and metastatic potential [35-37] and its target gene, oxidative stress protein, heme oxygenase 1 (HMOX-1)[38-41], a process that can be blocked by FABP4 inhibition. We also provide evidence of high FABP4 presence in experimental and patient prostate bone metastasis tissues and reveal the FABP4-PPARγ interaction as a potential driving mechanism of metastatic tumor growth in bone. Collectively, our data demonstrate that metastatic tumor cells utilize marrow adipocyte-supplied lipids to thrive and progress in skeletal sites and suggest a functional role for FABP4 abundance in the bone metastatic niche.

## RESULTS

### High fat diet promotes prostate tumor growth in bone

**Figure 1:**
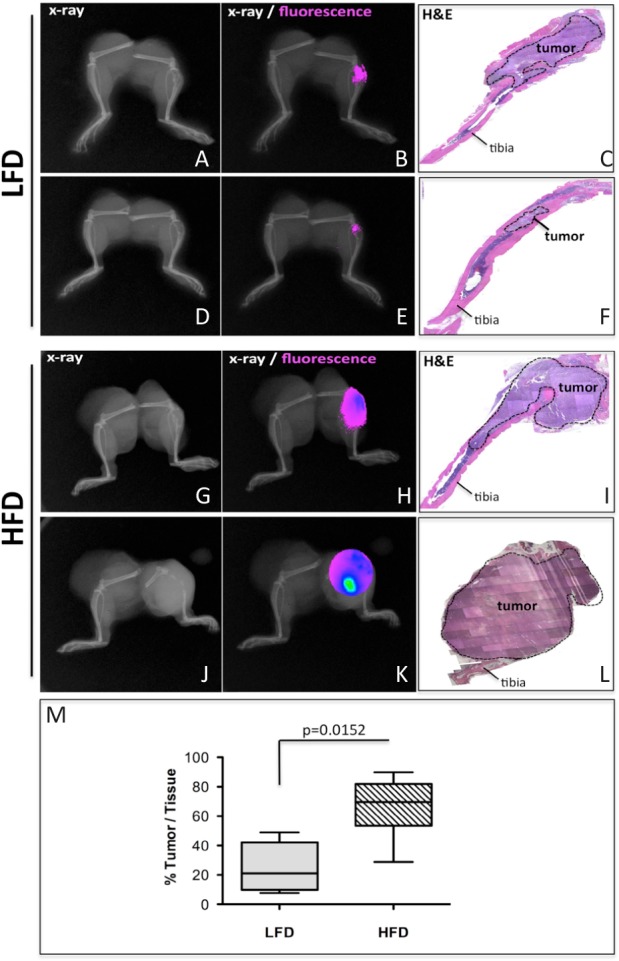
Diet-induced marrow adiposity accelerates progression of PC3 tumors in bone FVB/N/N5 Rag-1^−/−^ mice were fed normal (LFD, A-F) and high fat (HFD, G-L) diets for 8 weeks followed by intratibial injections of PC3-DsRed cells. The x-ray (A, D, G, J) and 600nm RFP fluorescence (B, E, H, K) imaging 6 weeks post injection (n=6 mice/group). C, F, I, L: H&E staining of tumor-bearing tibiae. M: Box and whisker plot showing percentage of tissue occupied by tumors. Results were analyzed by Mann-Whitney test.

**Figure 2:**
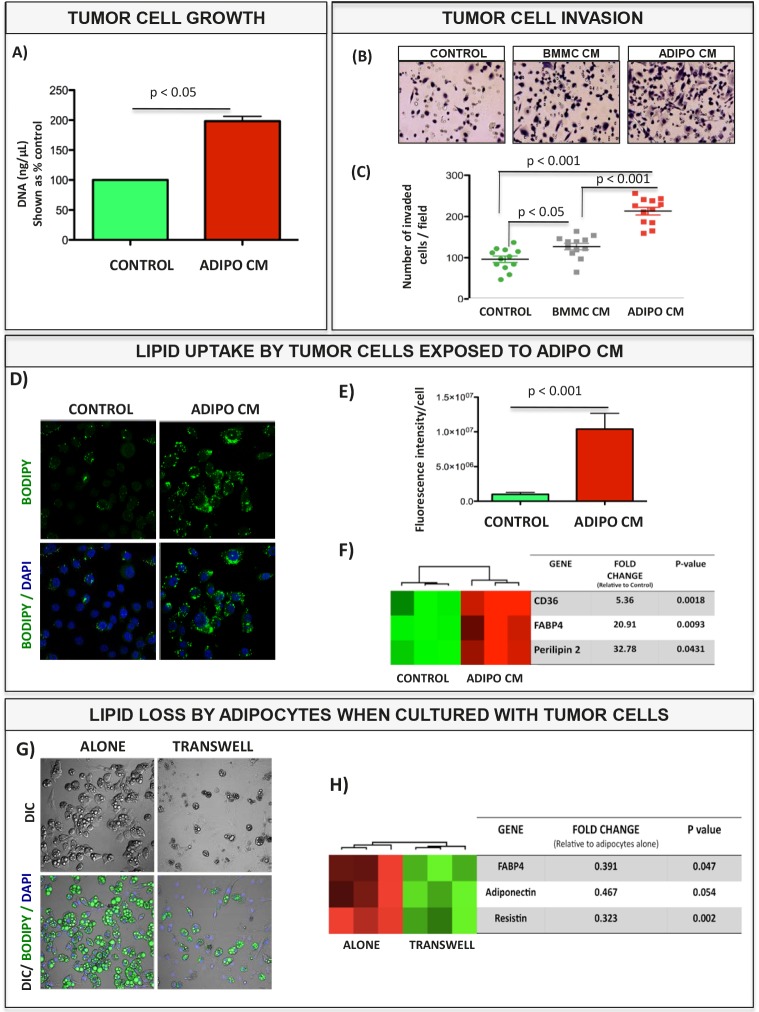
Bone marrow adipocyte-supplied lipids stimulate proliferation and invasion of prostate tumor cells and upregulate genes involved in fatty acid transport A: DNA assay results for cells grown in collagen I gels in the absence or presence of Adipo CM. B: Images of invasion filters coated with reconstituted basement membrane; cells in the absence (Control) or presence of media conditioned by Bone Marrow Mesenchymal cells (BMMC CM) or adipocytes (Adipo CM) were allowed to invade toward DMEM containing 10% FBS for 48 hours C: Quantitation results of invaded cells shown as % control ± SD. D: BODIPY 493/503 staining of lipid droplets (green) for control and Adipo CM-treated PC3 cells. E: Quantitation of lipid fluorescence (Metamorph). F: Taqman RT-PCR analysis (Life Technologies) of lipid droplet-associated genes: CD36, FABP4 and Perilipin 2 expression in PC3 cells +/− Adipo CM. Data are normalized to 18S. G: BODIPY 493/503 staining of lipid droplets (green) in adipocytes cultured alone (left panels) or in transwell with PC3 cells (right panels). H: Taqman RT-PCR analysis (Life Technologies) of adipocyte-specific gene (FABP4, Adiponectin and Resistin expression) in bone marrow adipocytes cultured alone or in transwell with PC3 cells. Data are normalized to HPRT1.

Growing literature evidence suggests that bone marrow adiposity is enhanced with increasing total body fat mass [12, 14, 22, 42, 43] and it correlates with induction of bone marrow inflammation [13, 14, 22]. Therefore, we investigated whether obesity-induced changes in the bone marrow in mice would have an impact on tumor progression in bone. As shown in Fig. 1, PC3-DsRed cells intratibially injected into mice fed high fat diet (HFD) grow strikingly larger than those injected into mice on normal (LFD) diet. In addition, bones of HFD mice prior to tumor implantation show profoundly increased marrow adiposity (Supplementary Fig. 1A and 1B). To investigate whether bone marrow fat cells play a direct role in these stimulatory effects on *in vivo* tumor growth, we isolated bone marrow mesenchymal cells (BMMC) from immuno-competent mice and differentiated them into adipocytes *in vitro* (Supplementary Fig. 2). Culture of prostate carcinoma cells (i.e., PC3 and C42B) and bone-trophic breast carcinoma cells (MDA 231 BO) in collagen I gels in the presence of adipocyte conditioned media (Adipo CM) for 60 hours resulted in significantly increased tumor cell proliferation as compared to cells grown under control conditions (Fig. 2A and Supplementary Fig. 3A). To examine the effects on invasiveness, tumor cells were exposed to serum-free media conditioned by BMMC cells (BMMC CM) or adipocytes (Adipo CM) for 24 or 48 hours. Exposure to Adipo CM significantly increased the number of PC3 cells capable of invading through reconstituted basement membrane-coated cell culture inserts after 48 hours (Fig. 2B–C) and as early as within 24 hours (Supplementary Fig.4). Similar results were obtained for prostate carcinoma C42B cells and bone-trophic breast carcinoma MDA 231BO cells (Supplementary Fig. 3B-G). Media conditioned by undifferentiated BMMC cells had no effect on invasion at 24 hours (Supplementary Fig.4), and induced only modest stimulatory effects at 48 hours (Fig. 2B–C), a result suggesting adipocyte-specific contribution to tumor invasiveness in the bone microenvironment. Notably, effects of either media on proliferation under serum-free conditions within the 24-48-hour timeframe of invasion assay were negligible (Supplementary Fig. 4C), suggesting that the observed increases in numbers of invaded cells were independent of growth-stimulatory effects of adipocyte-derived factors.

Growth- and invasion-promoting effects on tumor cells have been previously demonstrated for adipose tissue-derived adipocytes [24, 44, 45], particularly in the context of ovarian cancer, where they appear to serve as a source of energy-dense lipids [45]. Indeed, prostate and breast tumor cells exposed to bone marrow adipocyte-derived factors via Adipo CM (Fig. 2D, E, and Supplementary Fig. 5) or transwell co-culture (data not shown) exhibit cytoplasmic lipid accumulation and highly upregulate fatty acid transporter CD36, fatty acid chaperone FABP4, and lipid droplet marker perilipin 2 (Fig. 2F). These results strongly implicate lipid uptake by tumor cells in their increased growth and invasiveness in the presence of Adipo CM, result in agreement with previous findings by Brown *et al* [46]. Interestingly, when grown in a transwell co-culture with tumor cells, adipocyte numbers and their lipid content appear to be significantly reduced (Fig. 2G) as compared to adipocytes cultured alone. Associated with this decrease is a significantly lower expression of adipocyte-related genes such as adiponectin, FABP4 and resistin (Fig. 2H), a result in agreement with previously reported evidence of prostate cancer cells invasion and destruction of adipocytes within a primary human bone marrow stroma [46]. This indicates bi-directional interaction between tumor cells and adipocytes and suggests that by utilizing adipocyte-derived lipids, tumor cells may be affecting metabolic function of fat cells in the bone marrow.

### Bone marrow adipocytes induce FABP4, IL-1β and HMOX-1 in tumor cells

**Figure 3:**
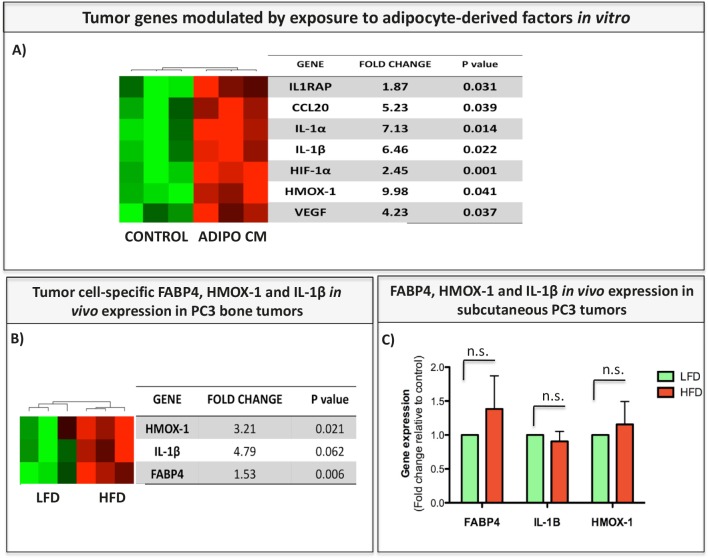
FABP4, IL-1β, and HMOX-1 are expressed in tumor cells exposed to adipocyte-derived factors *in vitro* and in bone tumors *in vivo* A: Genes upregulated in PC3 cells exposed to Adipo CM *in vitro* as detected by Taqman RT PCR Human Inflammation Array (Life Technologies). Data are normalized to HPRT1 and GUSB and shown as fold increase relative to control. B, C: Taqman RT-PCR of FABP4, IL-1β, and HMOX-1 in PC3 bone tumors (B) and PC3 subcutaneous tumors (C) from LFD and HFD mice. Data are normalized to HPRT1 and GUSB and shown as fold increase relative to LFD.

We next utilized commercially available Human Inflammation Taqman RT PCR array (Life Technologies) to explore other candidate genes modulated in tumor cells in response to adipocyte-derived factors. Since similar results were obtained when PC3 cells were grown in a transwell co-culture with adipocytes (data not shown), Adipo CM rather than transwell cultures was used as a source of adipocyte-supplied lipids for majority of experiments in this study (unless otherwise indicated). Upon exposure to Adipo CM, several genes implicated in inflammation and oxidative stress, particularly HMOX-1, interleukin 1 (i.e., IL-1α, IL-1β), CCL20, and HIF-1α were significantly upregulated (Fig. 3A). Among array-identified genes, three (i.e., FABP4, IL-1β, and HMOX-1) were also significantly induced in experimental PC3 bone tumors from HFD mice as compared to LFD mice (Fig. 3B). To determine if expression of these genes is driven specifically by bone marrow adiposity, we examined their levels in subcutaneous PC3 tumors from LFD and HFD mice. Not surprisingly, subcutaneous PC3 growth was significantly accelerated by chronic HFD (Supplementary Fig. 6), a result in line with previous reports on tumor-promoting effects of HFD in a number of cancer models, including LNCaP xenograft and TRAMP models of prostate cancer [47-49]. However, in contrast to skeletal tumors, and despite of HFD-enhanced growth and progression, expression of FABP4, IL-1β, and HMOX-1 in subcutaneous tumors did not appear to be modulated by HFD (Fig. 3C). This suggests expression of these factors by cancer cells in the bone marrow may be specifically important to tumor growth and progression in bone.

**Figure 4:**
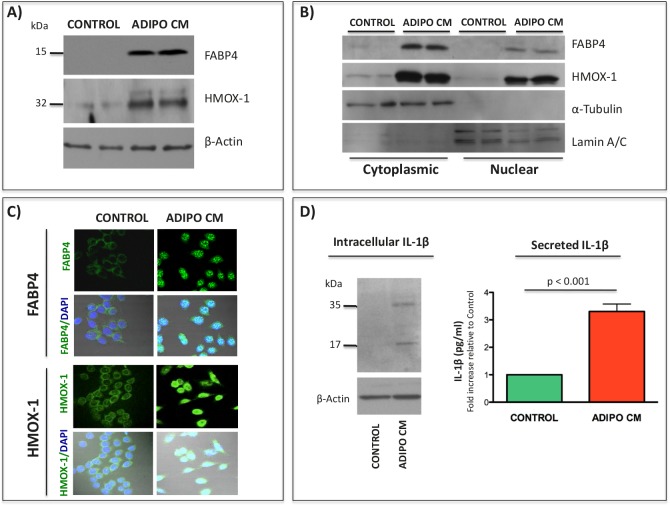
Induced expression of FABP4, HMOX-1 and IL-1β and nuclear translocation of FABP4 and HMOX-1 upon exposure of PC3 cells to Adipo CM A: Western blot for FABP4 (top), and HMOX-1 (middle) expression in PC3 cells grown in the absence or presence of Adipo CM. β-Actin was used as loading control (bottom). B: Cytoplasmic and nuclear fractions of PC3 cells probed for FABP4 (top) and HMOX-1 (top middle), cytoplasmic marker tubulin (bottom middle) and nuclear Lamin A/C (bottom). C: Immunofluorescence staining for FABP4 (top panels) and HMOX-1 (bottom panels) localization. Strong nuclear staining observed in Adipo CM-treated cells. D: Left panel: Immunoblot of intracellular IL-1β expression in PC3 cells. Pro-IL-1β (35 kDa) and active IL-1β (17 kDa) are detected upon exposure to Adipo CM. Right panel: IL-1β ELISA (R&D Systems) analysis of media conditioned by control and Adipo CM-treated PC3 cells. Data are normalized to DNA concentration in corresponding cell lysates and shown as fold increase in pg/ml relative to control.

**Figure 5:**
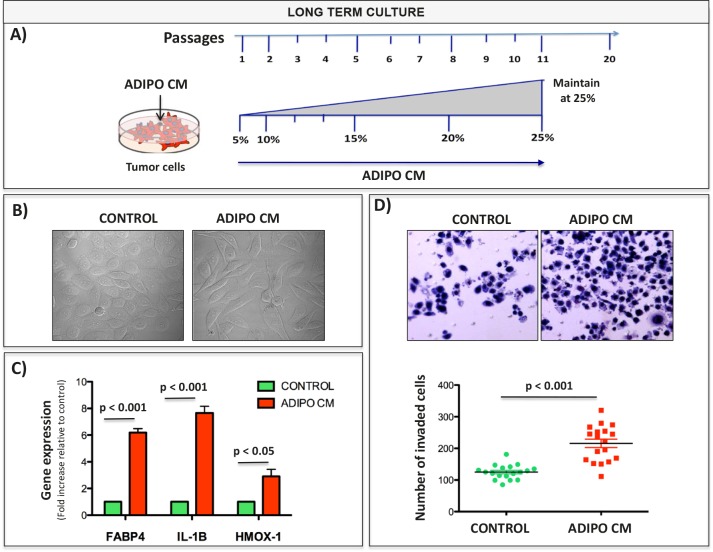
Chronic exposure to low dose Adipo CM increases invasiveness and induces FABP4, HMOX-1 and IL-1β expression A: Schematic of long-term culture conditions. Cells are exposed to gradually increasing concentration of Adipo CM (gradient of 5-25%) and maintained in 25% Adipo CM over multiple passages. B: DIC images showing invasive morphology of PC3 cells after chronic Adipo CM exposure. C: Taqman RT PCR for FABP4, IL-1β, and HMOX-1 expression of PC3 cells. Data are normalized to 18S and shown as increase relative to control cultures. D: Invasion assay of PC3 cells treated long-term with control or Adipo CM media for 12 passages. Prior to assay cells were serum starved, seeded in serum free media on top of rBM-coated filter and allowed to invade towards DMEM with 10% FBS for 48 hours. Top panels: Diff-Quik stained invasion filters. Bottom panels: Quantification results showing numbers of invaded cells.

Further investigations into adipocyte-driven FABP4, HMOX-1 and IL-1β expression in tumor cells revealed that transcriptional upregulation of these factors *in vitro* correlates with increased protein levels (Fig. 4A and Supplementary Fig. 7A). In addition to amplified levels, a cellular distribution of FABP4 and HMOX-1 changes from solely cytoplasmic to both cytoplasmic and nuclear (Fig. 4B, C). This is of importance as FABP4 is a known chaperone of lipids and regulator of nuclear signal transduction, particularly via PPARγ [27, 50, 51], while nuclear localization of HMOX-1 plays a role in regulation of oxidant responsive transcription factors [52]. In the case of IL-1β, an increase in intracellular levels of pro- and active forms of this cytokine is observed with Adipo CM treatments and correlates with increased secretion (Fig. 4D). To determine if FABP4, IL-1β, and HMOX-1 are involved in tumor cell adaptation to adipocyte-derived factors, we cultured PC3 cells in media containing gradually increasing amounts of Adipo CM (gradient of 5-25% over multiple passages) and then maintained them long-term in 25% Adipo CM. Cells cultured under chronic conditions had a spindle-shaped morhphology and were more invasive, a phenotype that persisted after short-term removal of Adipo CM (Fig. 5 and Supplementary Fig. 8). Notably, these cells maintained high expression of FABP4 and IL-1β, further strengthening the link between FABP4/IL-1β axis and tumor invasiveness.

### FABP4 is involved in tumor cell invasiveness

**Figure 6:**
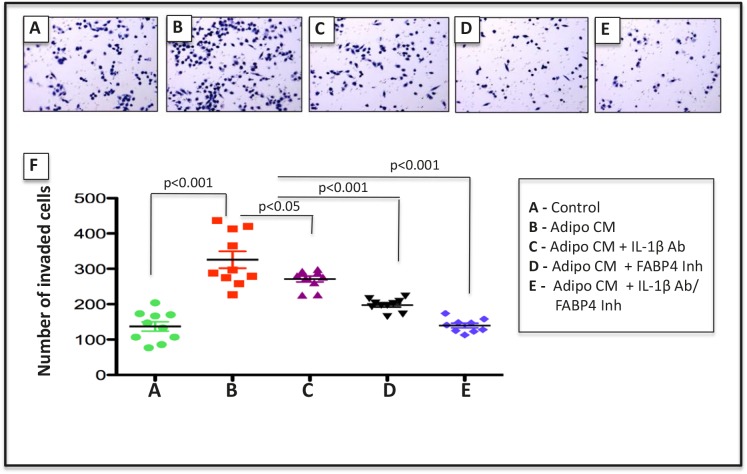
Blocking FABP4/IL-1β axis reduces invasiveness induced by Adipo CM Diff-Quik stained filters of Control (A), Adipo CM-treated (B), Adipo CM + IL-1β (C), Adipo CM+ FABP4 Inhibitor (D), and Adipo CM + Combination treatments (E). F: Numbers of invaded cells in response to each treatment. Data are representative of at least 3 experiments. P value < 0.05 is considered statistically significant.

Given the observed increases in FABP4 and IL-1β expression upon short- and long-term exposure to Adipo CM *in vitro* and with HFD *in vivo* we assessed the effects of FABP4 and IL-1β inhibition on invasion of PC3 cells stimulated with Adipo CM. A selective FABP4 inhibitor BMS309403 [53] very effectively inhibited Adipo CM-induced PC3 invasion, which was completely abolished when this compound was used in combination with IL-1β neutralizing antibody (Fig. 6). Importantly, neither of the treatments affected tumor cell proliferation (data not shown), demonstrating that inhibitory effects on invasion were not due to growth inhibition or toxicity.

### FABP4 is expressed in experimental and patient bone metastatic lesions

**Figure 7:**
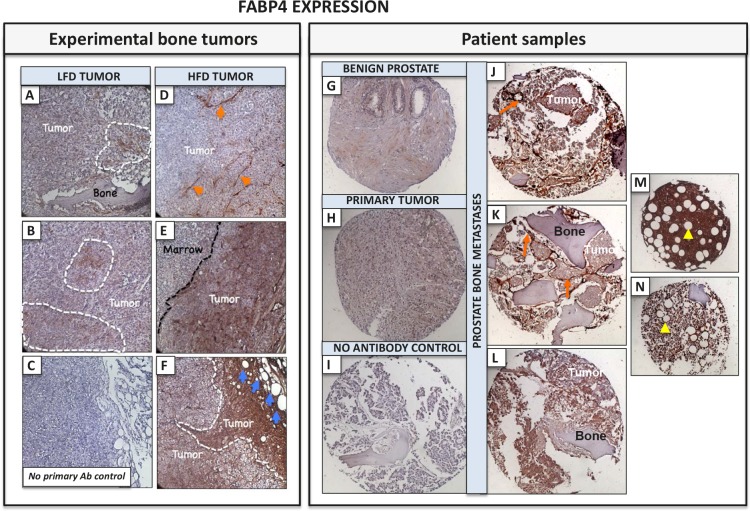
FABP4 is strongly expressed in experimental PC3 bone tumors from HFD mice and in bone metastatic tissues from prostate cancer patients Immunohistochemical analysis of FABP4 expression (Nova Red). A-B: Tumor sections from LFD mice; areas of the tumor with FABP4 positivity indicated by dotted line; C: No primary antibody control; D-F: sections from HFD mice. FABP4 expressed in tumor blood vessels (D, orange arrows); and tumor cells (E), especially in the areas neighboring adipocytes (F, blue arrows); 20 x images. FABP4 immunostaining of TMA sections from normal prostate (G), primary prostate tumor (H), and bone metastatic lesions (J-L). FABP4 expression detected in tumor cells, bone marrow cells and particularly strongly in blood vessels surrounding tumor cells (orange arrows). I: no antibody control. M, N: sections of adipocyte-rich bone marrow strongly positive for FABP4 (yellow arrows).

In an effort to explore localization of FABP4 in the bone tumor microenvironment, we performed immunohistochemical analyses of PC3 bone tumors from LFD and HFD mice. LFD tumors exhibited very modest FABP4 expression (Fig. 7A–B), whereas strong FABP4 presence was detected in HFD tumors (Fig. 7D–F). FABP4 was expressed primarily in endothelial cells (Fig. 7D) as shown previously in ovarian cancer [25], tumor cells colonizing the bone marrow (Fig. 7E), bone marrow adipocytes, and particularly strongly in tumor cells neighboring adipocyte-rich areas (Fig. 7F). Immunohistochemical findings from experimental bone tumors were then confirmed in tissue samples from prostate cancer patients, which showed FABP4 is strongly localized to endothelial cells (Fig 7J, K) but it is also highly present in tumor cells (Fig. 7J–L). The most abundant FABP4 expression was detected in bone tissue cores infiltrated with high numbers of bone marrow adipocytes, where both the adipocytes and the surrounding bone marrow cells were highly FABP4-positive (Fig. 7M, N). FABP4 positivity in bone tumor lesions was much higher than in benign prostate and primary tumor samples (Fig. 7G, H and Supplementary Fig. 9), indicating there might be a potential functional reason for its abundance in the bone metastatic niche.

### Upregulation of FABP4/IL-1β/HMOX-1 axis is PPARγ-dependent

**Figure 8:**
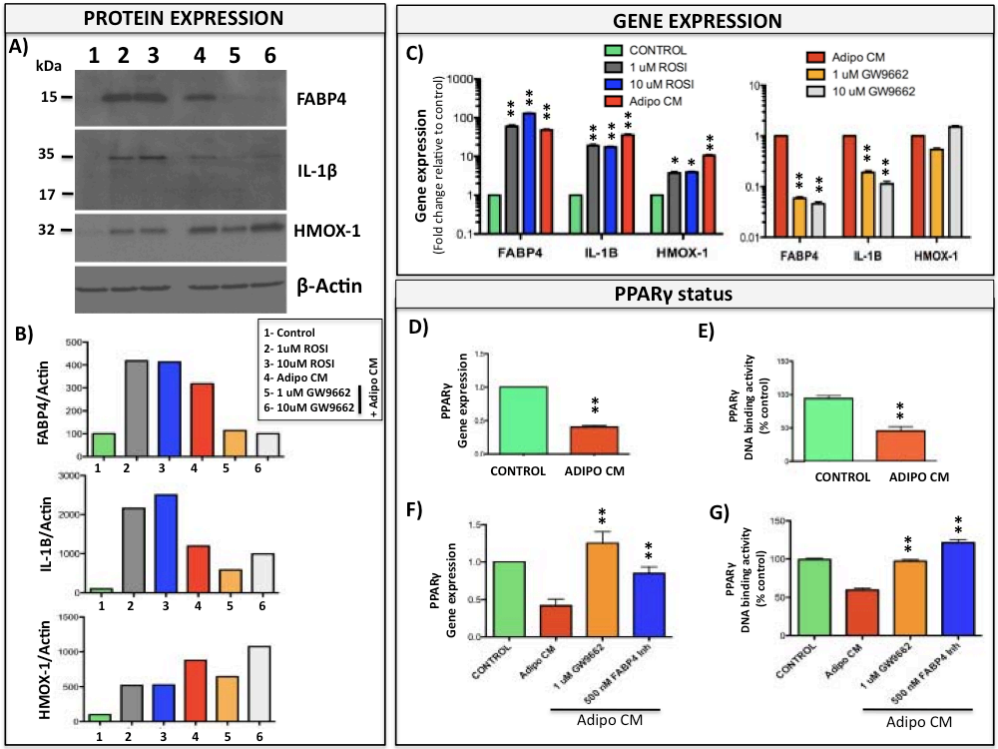
PPARγ is involved in Adipo CM-induced expression of FABP4, IL-1β, and HMOX-1 A: Western blot analysis of intracellular FABP4, IL-1β and HMOX-1 expression in PC3 cells grown in the absence or presence of rosiglitazone (ROSI, 1 and 10μM), Adipo CM, and Adipo CM in the presence of PPARγ antagonist GW9662 (1 and 10μM). B: Densitometric analysis of FABP4 (top panel), IL-1β (middle panel) and HMOX-1 (bottom panel) bands normalized to β-actin. C: Taqman RT PCR analysis of FABP4, IL-1β and HMOX-1 expression in PC3 cells grown in the absence or presence of rosiglitazone (1 and 10μM), Adipo CM, and Adipo CM in the presence of PPARγ antagonist GW9662 (1 and 10μM). Similar increase in FABP4, IL-1β and HMOX-1 expression is observed with ROSI and Adipo CM and expression of FABP4 and IL-1β is reduced with GW9662. D: PPARγ gene expression (Taqman RT PCR) in PC3 cells exposed to Adipo CM. Data are normalized to 18S and GUSB and show a decrease in PPARγ levels with Adipo CM; E: DNA binding assay in nuclear fractions from control and Adipo CM-treated PC3 cells showing reduced PPARγ DNA binding upon Adipo CM treatment. Adipo CM-suppressed gene expression (F) and DNA-binding activity (G) of PPARγ can be restored by GW9662 antagonist or FABP4 inhibitor; Values * p <0.05; ** p <0.01 are considered statistically significant.

It is well-established that FAPB4 is one of the PPARγ target genes and its expression in adipocytes positively correlates with PPAR activation [29, 54]. To determine if PPARγ is also involved in FABP4 overexpression in tumor cells exposed to adipocyte-derived factors, we treated tumor cells with either PPARγ agonist rosiglitazone (ROSI) or with Adipo CM in the absence or presence of PPARγ antagonist GW9662. FABP4 levels were induced to comparable levels by ROSI and Adipo CM treatments and were abolished by GW9662 in PC3 (Fig. 8A, B) and ARCaP(M) cells (Supplementary Fig. 10), suggesting FABP4 expression in these cells is indeed PPARγ-driven. IL-1β and HMOX-1 levels were also increased by ROSI and Adipo CM treatments; however, the HMOX-1 expression was not affected by the GW9662 antagonist (Fig. 8A, B). Changes in FABP4, IL-1β and HMOX-1 protein were mirrored by the similar changes in gene expression levels (Fig. 8C) and were observable as early as 2 hours after exposure to Adipo CM or ROSI (Supplementary Fig. 11A-C). Interestingly, cells exposed to Adipo CM showed significant reduction in the mRNA levels and the DNA binding activity of PPARγ (Fig. 8D, E, and Supplementary Fig. 11D) that could be restored by either GW9662 antagonist or FABP4 inhibitor (Fig. 8F, G). Given the fact that FABP4 is known to regulate the transcriptional activity of PPARγ by delivering nuclear ligands [30], it is possible that observed PPARγ downregulation is due to overstimulation of the receptor by the abundance of FABP4-transported ligands.

## DISCUSSION

Adipocytes are active and important modulators of tumor microenvironment, either through secreted adipokines or direct contact with neighboring cells [24, 25, 55, 56]. Their impact is particularly visible in cancers that grow in adipocyte-rich microenvironments such as the breast, or tumors which metastasize to fat cell-enriched sites such as gastric and ovarian cancers [56]. In these tumors, adipocyte-cancer cell cross-talk has metabolic implications for both interacting cell types: it stimulates mitochondrial metabolism in cancer cells due to high-energy lipid transfer, and induces metabolic changes in adipocytes resulting in enhanced lipolysis [25, 56]. Consequently, these events can trigger microenvironmental changes that support tumor growth and survival. Several studies have positively linked adipocytes, especially those within visceral and periprostatic adipose tissues, to prostate cancer progression and poor prognosis [56]. Little is known, however, how interactions of these metabolically active cells with prostate cancer cells impact tumor growth and behavior in the bone metastatic niche. It is well established that an increase in marrow fat content due to age and obesity compromises bone integrity and function [7, 8, 13]. In fact, considerable research has been dedicated to studying effects of marrow adiposity on bone health, especially in the context of osteoporosis [6, 8, 57, 58]. Age and obesity are also known to greatly increase the incidence of skeletal metastases and death from prostate cancer [1-5]; however, no studies to date have directly addressed the role of marrow fat cells in tumor cell adaptation and growth in the skeleton.

Herein, using diet-induced obesity (DIO) model, a documented approach to induce significant marrow adiposity [8, 13, 14], we demonstrated that bone marrow fat cells have promoting effects on growth and progression of prostate tumors in bone. It has been previously postulated that increased invasiveness of prostate cancer cells in the bone marrow is driven by adipocyte-derived lipids from nearby marrow fat cells [20]. Here, we provided specific evidence for accumulation of marrow fat cell-supplied lipids by tumor cells, and demonstrated that the accelerated growth and invasiveness involves upregulation of tumor-derived FABP4, IL-1β and HMOX-1. We focused on FABP4 in particular because this lipid chaperone was strongly expressed in bone metastatic lesions from prostate cancer patients and in bone tumors, but not subcutaneous tumors from HFD mice, and importantly, its inhibition was sufficient to abrogate adipocyte-induced tumor cell invasion. This indicates FABP4 is involved in modulating growth and behavior of metastatic tumor cells by increasing the availability of energy-dense lipids, as shown recently in ovarian cancer cells where its upregulation was linked to activation of β-oxidation pathway [25]. Since β-oxidation pathway is already a predominant source of energy for prostate cancer cells at the primary site [59], it is likely that the metastatic cells utilize this pathway to sustain their growth in bone. To thrive at the secondary site, such as bone, tumor cells have to adapt their metabolism to take advantage of available sources of energy provided by the host cells. They are also likely to induce metabolic changes in bone marrow adipocytes, a phenomenon recently defined as “two-compartment tumor metabolism” [60] or “metabolic symbiosis” [61]. Whether we can use tumor-adipocyte co-dependence to therapeutically starve metastatic cancer cells in the bone marrow niche emerges as an attractive concept that warrants further studies.

As a mediator of lipid trafficking between adipocytes and tumor cells, FABP4 itself may be a viable candidate for therapeutic targeting. Important to FABP4 expression and activity is its interaction with PPARγ. FABP4 is induced by free fatty acid (FFA) influx into the cells, its expression is regulated by PPARγ, but at the same time, transcriptional activity of PPARγ is driven by nucleocytoplasmic shuttling of FABP4 [30]. Ligand delivery and binding can both activate the PPARγ receptor and stimulate its destruction [62]. In the present study, exposure of tumor cells to adipocyte-derived factors led to PPARγ-driven FABP4 upregulation followed by PPARγ downregulation. This could be due to increased ligand delivery by FABP4 resulting in receptor overstimulation, or due to FABP4–mediated activation of JNK/MAPK pathways [27] and MEK/ERK-mediated PPAR phosphorylation, established mechanisms of PPARγ inactivation [63]. In either case, PPARγ suppression is likely to result in loss of metabolic oversight and consequent malignant transformation [32]. Decreased PPARγ signaling due to HFD was recently associated with reduced androgen signaling and low grade PIN (Prostatic Intraepithelial Neoplasia) [31]; however, PPARγ's status has not been previously investigated in a context of marrow adiposity and metastatic prostate cancer. Data presented in this study suggest that co-dependent interaction of this receptor with FABP4 may be a potential mechanism of tumor adaptation to the bone metastatic niche. In addition to its expression in adipocytes and tumor cells, FABP4 localizes strongly to endothelial cells and bone marrow cells, important components of bone metastatic niche. Notably, FABP4 is a reported target of VEGF/VEGFR2 axis [64], has been implicated in angiogenesis associated with glioblastoma aggressiveness [65], and its deficiency in mice has inhibitory effects on ovarian tumor vasculature [25]. In the present study, tumor cells exposed to adipocyte-derived factors overexpress VEGF (Figure 3A). This suggests a potential link between FABP4-driven fatty acid uptake and VEGF expression by tumor cells that may be a contributing mechanism to tumor progression in bone that needs further investigation.

It is noteworthy that in addition to its effects on bone tumors, diet-induced obesity accelerated subcutaneous tumor growth in HFD mice, a result indicating potential systemic consequences of the diet itself. This finding is in line with the emerging, although still debated, roles of dietary lipids in prostate cancer development and progression [66, 67]. Although our study did not directly address the dietary effects on bone metastatic niche, our data strongly implicated bone marrow adipocytes in metastatic tumor growth in bone. Our *in vivo* results identifying FABP4, IL-1β and HMOX-1 as factors influencing invasiveness and tumor growth in the bone but not in the subcutaneous site were recapitulated using bone marrow adipocyte cultures *in vitro*. Future studies utilizing genetic models of obesity and age-induced models of marrow adiposity will provide more detailed understanding of molecular mechanisms behind marrow adipocyte involvement in modulating tumor behavior in bone.

Bone marrow adipocytes are very much under-investigated and under-appreciated components of bone tumor microenvironment. Our results suggest that they may be a major source of energy for the metastatic tumor cells in bone. To our knowledge, this is the first study demonstrating the importance of lipid trafficking in progression of prostate cancer in bone and implicating FABP4 as a mediator of adipocyte-tumor cells interactions within metastatic niche. Understanding how marrow adipocytes influence prostate tumor metabolism may reveal unique therapeutic targets and treatment opportunities for this incurable metastatic disease.

## MATERIALS AND METHODS

### Materials

Dulbecco's modified Eagle's medium (DMEM), RPMI-1640 medium, and other chemicals, unless otherwise stated, were obtained from Sigma (St. Louis, MO). HyClone fetal bovine serum (FBS) was from ThermoFisher (Pittsburg, PA). T-Medium, Trypsin–EDTA, collagenase, Alexa Fluor 488–conjugated donkey anti-goat and anti-rabbit IgG, rabbit anti human/mouse FABP4 and Gentamicin (G418) were from Invitrogen (Carlsbad, CA). PureCol® collagen type I was from Advanced Biomatrix (San Diego, CA). Mouse monoclonal antibody to lamin A+C was from Abcam (Cambridge, MA). Mouse monoclonal E7 Beta tubulin antibody was from Developmental studies Hybridoma Bank (Iowa City, IA). StemXVivo Adipogenic Suppliment, Goat anti-human/mouse HMOX-1, goat anti human IL-1β antibodies and human IL-1β ELISA kits were from R&D Systems (Minneapolis, MN). Rabbit anti-human/mouse ß-actin antibodies were from Novus Biologicals (Littleton, CO) and Rosiglitazone and PPARγ Transcription factor assay kit were from Cayman Chemical Company (Ann Arbor, MI). RNeasy Mini Kits were from Qiagen (Valencia, CA). Immunoblotting “Western Lightning ECL Plus” detection kits were from Perkin Elmer LLC (Waltham, MA). Z-Fix was from Anatech, LTD (Battle Creek, MI). ImmPRESS Anti-Goat and Anti-Rabbit IgG Peroxidase Polymer Detection kits and NovaRED kit for peroxidase, were purchased from Vector Laboratories (Burlingame, CA).

### Cell Lines

PC3, an androgen-independent osteolytic cell line derived from a bone metastasis of a high-grade adenocarcinoma [68], was purchased from American Type Culture Collection (ATCC; Manassas, VA). The ARCaP(M), an Androgen-Repressed Metastatic Prostate Cancer Cells M (‘Mesenchymal’ Clone) [69] were purchased from Novicure Biotechnology (Birmingham, AL). The human prostate cancer C4-2B cell line was kindly provided by Dr. Leland W. K. Chung, Emory University (Atlanta, GA). The MDA-231BO is a bone-seeking clone derived from MDA MB-231 breast carcinoma cells [70] and was kindly provided by Dr. Toshiyuki Yoneda (University of Texas Health Science Center, San Antonio, TX). The PC3-DsRed cell line was established by stable transfection with pDsRed2-N1 vector (Clontech Laboratories, Palo Alto, CA), as described previously [42]. PC3-DsRed and MDA-231BO cells were grown in DMEM medium with 10% FBS, C4-2B cells were grown in RPMI-1640 medium with 10% FBS, and ARCaP(M) cells in T-medium with 5% FBS. Cells were maintained in a 37°C humidified incubator ventilated with 5% CO_2_.

Primary mouse bone marrow stromal cells (mBMSC) were isolated from femurs and tibiae of 6- to 8- week old FVB/N mice according to previously established protocols [71]. To induce bone marrow adipocyte differentiation, mBMSC cells were plated in 3D collagen I gels, grown to confluency for 48-72 hours and treated with adipogenic cocktail (30% StemXVivo Adipogenic Suppliment, 1 μM insulin, 2 μM Rosiglitazone; DMEM and 10% FBS) for 8-10 days. Differentiated bone marrow adipocyte cultures were washed 3 times with PBS and cultured in serum-free DMEM for 12-16 hours. Serum-free conditioned media was collected, centrifuged, aliquoted and stored at −80°C. Unless otherwise stated, prior to use, serum-free medium collected from adipocyte cultures was diluted 1:1 with serum-free culture medium appropriate for each cell line and designated ‘Adipo CM’.

### Animals

All experiments involving mice were performed in accordance with the protocol approved by the institutional Animal Investigation Committee of Wayne State University and NIH guidelines. *In vivo* xenograft studies were performed in male mice in the FVB/N background with homozygous null mutation in the Rag1 gene [FVB/N/N5, Rag-1^−^/^−^]. All mice were bred in-house.

### Diets

At 5 weeks of age, mice caged in the groups of 4 were started on either a low-fat (LFD) diet (10% calories from fat; Research Diets no. D12450Bi) or a high-fat (HFD) diet (60% calories from fat; Research Diets no. D12492i). D12450Bi is a standard matched control diet for D12492i as recommended by Research Diets (http://www.researchdiets.com/search/collection1?q=D12492). Mice were maintained on respective diets for 8 weeks prior to and 6 weeks following the tumor implantation into bone or subcutaneously (total of 14 weeks) and the study was repeated two more times.

### Tissue Microarrays (TMAs)

Tissue microarrays containing benign, primary, and metastatic prostate cancer tissue sections were collected through a Rapid Autopsy program at the University of Michigan [27] and were a kind gift of Dr. Kenneth Pienta. Detailed histopathologic evaluation was performed by Wayne State University/Karmanos Cancer Institute pathologist, Dr. Dong Ping Shi.

### Intratibial injections of prostate cancer cells

Intratibial tumor injections were performed under isoflurane inhalational anesthesia according to the previously published procedures [42]. Briefly, a cell preparation containing 5 ×10^5^ of PC3-DsRed cells in PBS (20 μl, right tibia), or PBS alone (control, 20 μl, left tibia) was injected into the bone marrow. Six weeks post-injection mice were euthanized, and control and tumor-bearing tibiae were removed and imaged *ex vivo*. Fluorescence and x-ray images were obtained using a Carestream XVivo Multimodal Animal Imager. For microenvironmental control, separate groups of LFD and HFD mice were injected subcutaneously with 50 μl of PC3-DsRed cell suspension (5 ×10^5^ cells in PBS/Cultrex). Half of the intratibial tumor samples from each group and half of each subcutaneous tumor were fixed in Z-fix, bone tumors were decalcified, and all samples were embedded in paraffin. Remaining tissues were snap-frozen in liquid nitrogen, powderized using a tissue pulverizer and RNA was isolated using Trizol and RNeasy Mini Kit.

### Bone histomorphometry and adipocyte quantification in experimental bone tumors

The 5μm longitudinal sections from tibiae were deparaffinized, and stained with hematoxylin and eosin (H&E) as described previously [42, 71]. Digital images were captured under 10x magnification using a Zeiss Axioplot microscope with CCD camera (Zeiss, Gottingen, Germany) and ImageJ software was used to calculate the percentage of the area occupied by the tumor [42]. In addition, adipocyte numbers in tibiae of LFD and HFD mice were determined from H&E images, expressed as number of adipocytes per area of bone marrow and represented as Box and Whisker plots.

### FABP4 immunohistochemistry

Tumor-bearing tibiae from LFD and HFD mice and patient TMA sections were analyzed by immunohistochemistry for expression and localization of FABP4 (rabbit anti-human/mouse FABP4; 1:300). ImmPRESS Anti-Goat Peroxidase Polymer Detection systems along with a NovaRED kit as a substrate were used for the peroxidase-mediated reaction.

### Tumor cell proliferation

DNA concentrations in cell lysates were determined by the method of Downs and Wilfinger as previously described [71-73]. To determine tumor cell proliferation under conditions of invasion assays, Vybrant® MTT Cell Proliferation Assay kit (Life Technologies) was used. Conversion of MTT (3-(4,5-dimethylthiazol-2-yl)-2,5-diphenyltetrazolium bromide) to formazan by viable tumor cells was measured at 540 nm according to manufacturer's instructions.

### Invasion assays

Tumor cells were serum starved for 3 hours or overnight and seeded on top of the BD invasion filter (8 μm pore size) coated with 3-D Culture Matrix™ reduced growth factor basement membrane extract (Trevigen, 1mg/ml). Cells were seeded at the density of 5 × 10^4^ cells/filter in serum-free medium (Control) or Adipo CM. To study effects of FABP4 and IL-1β inhibition on tumor invasion 500 nM FABP4 inhibitor (BMS309403[53]; Calbiochem) or IL-1β neutralizing antibody (R&D Systems; 1μg/ml) were added to Control or Adipo CM. Media containing 10% FBS was added to the bottom chamber as chemoattractant. Cells were allowed to invade for 48 hours and filters were stained with Diff-quick reagents. Invading cells were visualized using a Zeiss Axioplot microscope, and counted using ImageJ software. Data were collected from at least three independent experiments performed in triplicate.

### Short- and long-term treatment with Adipo CM

For short-term conditioned media treatments, tumor cells were seeded on collagen I-coated 60 mm dishes (at the density of 5 × 10^5^cells/dish) or on 6-well plates (at the density of 2 × 10^5^ cells/well) in DMEM containing 5% FBS in the absence (Control) or presence of Adipo CM. For indirect adipocyte-tumor cell co-cultures, mBMSC cells were seeded in collagen I-coated 6-well plates, differentiated into adipocytes, and tumor cells were seeded on top of a Transwell filter (0.2 μm pore size) to allow sharing of soluble factors between the two cell types. Cells were cultured as described for 48 hours and serum-starved for additional 12-16 hours prior to sample collection for analyses. For experiments assessing chronic exposure to Adipo CM, cells were grown in control medium (5% FBS) or medium containing gradually increasing amounts of Adipo CM (gradient of 5-25% Adipo CM; 5% FBS) over 12 passages and maintained in 25% Adipo CM up to 20 passages. For protein analyses, lysates were re-suspended in SME buffer [42, 71], media were concentrated through 3K Millipore centrifugal filters, and all samples were stored at −80°C for future use. For RT PCR analyses, cells were collected into RLT buffer and RNA purified using RNeasy Mini Kit. Extraction and separation of cytoplasmic and nuclear protein fractions was performed using NE-PER Nuclear and Cytoplasmic Extraction kit (Thermo Scientific) according to manufacturer's instructions. Protein content in each fraction was determined using BCA protein kit (ThermoScientific).

### Immunofluorescence analyses

Cytoplasmic and nuclear HMOX-1 and FABP4 were examined by immunofluorescence staining using goat anti-human HMOX-1 (1:50), rabbit anti-human/mouse FABP4 (1:200) antibodies, and Alexa 488 secondary antibodies. Fluorescent images were captured with a Zeiss LSM510 META NLO confocal microscope using 40x oil immersion lens. Controls were run in the absence of primary antibodies.

### TaqMan RT-PCR analyses

The cDNA from cells and *in vivo* samples was prepared from 2 μg of total RNA using High-Capacity cDNA Reverse Transcription kit (Applied Biosystems). The analyses of genes associated with lipid uptake were performed using TaqMan® Individual Gene Expression assays for FABP4 (Hs010086177), CD36 (Hs01567185), and Perilipin 2 (Hs00605340). Assays were done on three biological replicates using TaqMan® Fast Universal PCR Master Mix and 50 ng of cDNA/well and all reactions were run on an Applied Biosystems StepOnePlus™ system. For initial screening of genes modulated by Adipo CM, TaqMan® Array 96 –well Fast Plates (Human Inflammation) were used. Three biological replicates of each sample were pooled together and assays were run in duplicate. Results for human HMOX-1 (Hs00157965), PPARγ (Hs0115513) and IL-1β (Hs00174097) were then validated on triplicate samples with TaqMan® Individual Gene Expression Assays. The same assays (FABP4, HMOX-1, and IL-1β) were performed on triplicate samples of PC3 bone tumors from LFD and HFD mice. For all human genes, data were normalized to beta-glucuronidase (GUSB; Hs99999908) and hypoxanthine phosphoribosyltransferase (HPRT1; Hs 99999909). For assessment of adipocyte-specific genes in adipocytes grown in co-culture with tumor cells following murine Taqman assays were used: FABP4 (Mm00445878), Adiponectin (Mm00456425) and Resistin (Mm00445641). Data were normalized to HPRT1 (Mm00446968). DataAssist™ Software (Applied Biosystems) was used for all analyses.

### Immunoblot analyses

Lysate and media samples were loaded based on DNA concentrations in the corresponding lysates and proteins were electrophoresed on 12% or 15% SDS-PAGE gels, transferred to PVDF membranes and immunoblotted for HMOX-1 (1:1,000), FABP4 (1:500), IL-1β (1:500), and β-actin (1:5,000). Tubulin (1:2000) and lamin A/C (1:500) were used for purity assessment and normalization of cytoplasmic and nuclear fractions. All horseradish peroxidase-labeled secondary antibodies were used at 1:10,000. Quantification and analyses of bands were performed using a Luminescent Image Analyzer LAS-1000 Plus (Fujifilm, Stamford, CT) and expressed as arbitrary units (AU) per square millimeter.

### Statistical analyses

Data were presented as mean ± SD and statistically analyzed using student T-test. For three or more groups, one-way analysis of variance was used. Box and whisker plots were used and Mann-Whitney tests applied for data following non-parametric distribution.

## Supplementary Figures


