# Supplementary material for: Bone marrow adipocytes promote tumor growth in bone via FABP4-dependent mechanisms
Source: Oncotarget. 2013 Oct 29;4(11):2108–23. doi: 10.18632/oncotarget.1482 (PMC3875773; doi:10.18632/oncotarget.1482)
Supplement: Supplementary file 1 [file oncotarget-04-2108-s001.pdf]

Bone Marrow Adipocytes Promote Tumor Growth in Bone via FABP4-dependent Mechanisms - Herroon et al

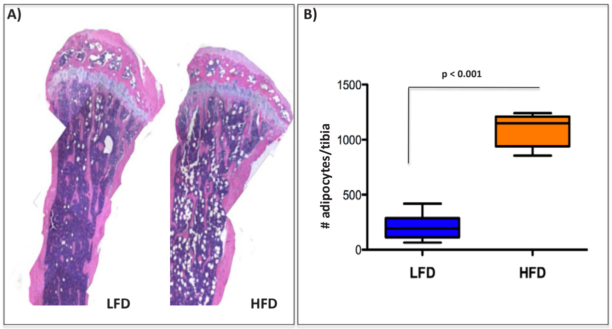

Supplementary Figure 1: Bone marrow adiposity is induced by chronic HFD in mice. A: H&E staining of tibiae sections from LFD (left) and HFD (right) mice. B: Quantitation of adipocyte numbers in tibia of LFD and HFD mice. Data are shown as number of fat cells/tibiae section +/- S.D (n=6).

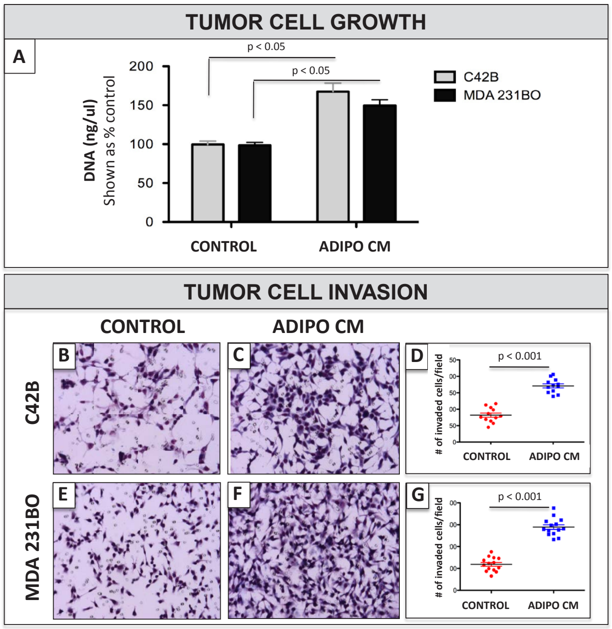

Supplementary Figure 3: Adipocyte-derived factors stimulate growth and invasion of C4-2B prostate and MDA-231BO breast carcinoma cells. A: DNA assay results for C4-2B and MDA-231BO cells grown in collagen I gels in the absence or presence of Adipo CM. B, C, E, F: Images of invasion filters coated with reconstituted basement membrane; cells were allowed to invade in for 48 hours in the absence (B, E, control) and presence of Adipo CM (C, F). D, G: Quantitation results of invaded cells shown as % control ± SD.

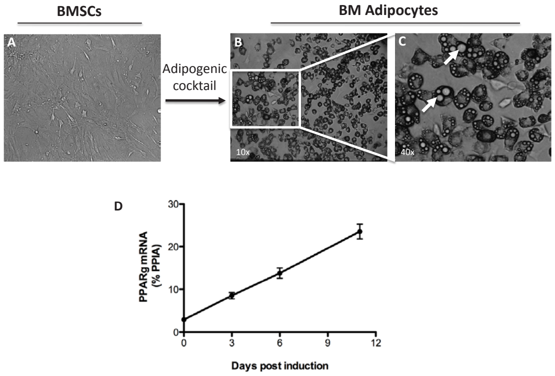

Supplementary Figure 2: In vitro differentiation of bone marrow stromal cells (BMSC) into adipocytes. A: DIC image of undifferentiated BMSCs prior to adipogenic cocktail treatment. B: Bone marrow adipocytes; Magnification, 10x. C: 40x image of selected area from the 10x image. D: PPARγ mRNA expression in bone marrow cells upon adipogenic cocktail treatments; increasing expression indicates adipocyte maturation; expression levels were measured on day 0, 3, 6 and 11 of treatment and normalized to Peptidylpropyl isomerase A (PPIA).

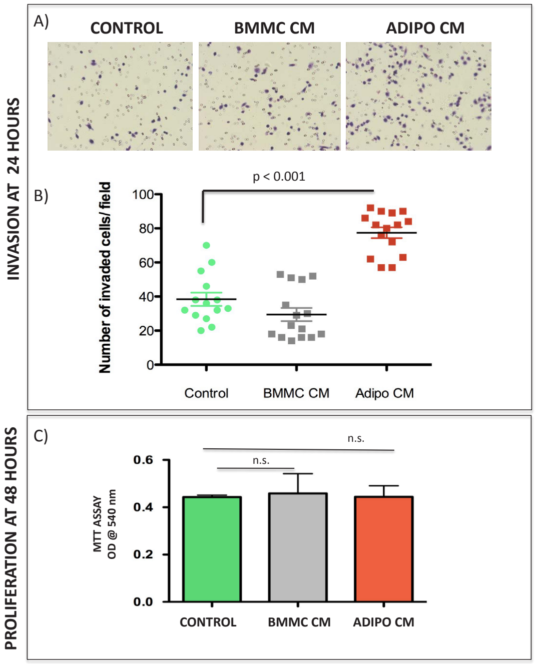

Supplementary Figure 4.: Adipocyte-conditioned (Adipo CM), but not BMMC-conditioned medium (BMMC CM) stimulates PC3 invasion after 24 hours. A: Images of invasion filters coated with reconstituted basement membrane; cells were allowed to invade in for 24 hours in the absence (control; left panel) or presence of BMMC CM (middle panel), or Adipo CM (right panel). B: Quantitation results of invaded cells shown as % control ± SD. C: MTT assay results showing effects of control, BMMC CM and Adipo CM on proliferation of PC3 cells under serum-free conditions at 48 hours. Data are shown as mean +/- SD (n= 6 replicates).

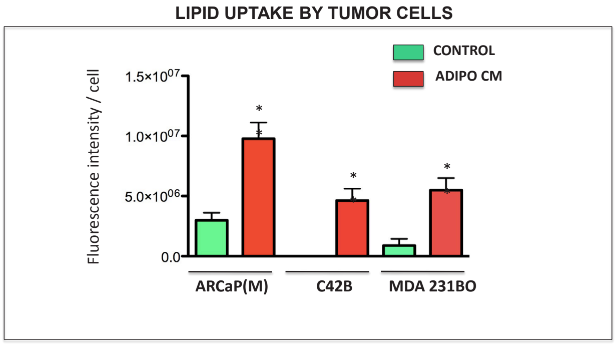

Supplementary Figure 5: Uptake of adipocyte-supplied lipids by tumor cells. Quantitation of lipid fluorescence (Metamorph) based on BODIPY 493/503 staining of lipid droplets (green) for control and Adipo CM-treated ARCaP (M) C42B and MDA 231BO cells. Data are shown as fluorescence/ cell +/- SD (n=6).

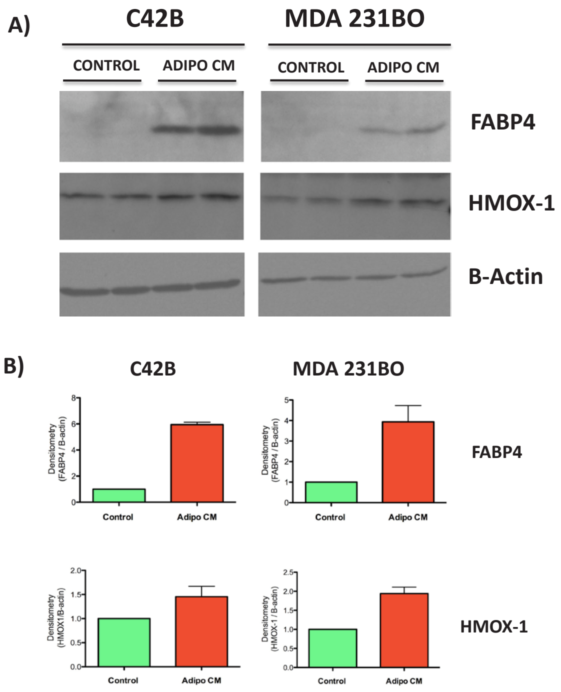

Supplementary Figure 7: Expression of FABP4 and HMOX-1 is induced by Adipo CM in C4-2B prostate carcinoma and MDA-231BO breast carcinoma cells. A: Western blot for FABP4 (top), and HMOX-1 (middle) expression in C4-2B (left) and MDA-231BO cells (right) grown in the absence or presence of Adipo CM. β-Actin was used as loading control (bottom). B: Densitometric analysis of FABP4 (top panels) and HMOX-1 (bottom panels) bands normalized to β-actin.

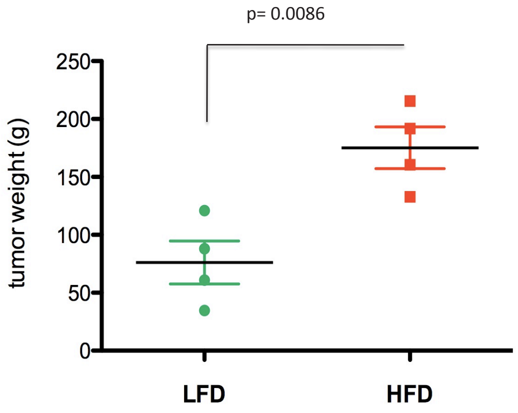

Supplementary Figure 6: Chronic HFD accelerates growth of subcutaneous PC3 tumors. PC3 cells (5 x 105) were injected subcutaneously into the flank of Rag-1-/- mice and allowed to grow for 5 weeks. Ex vivo tumor weight was measured in grams. PC3 tumors from HFD mice were significantly larger than tumors from LFD mice; n=4 mice/group.

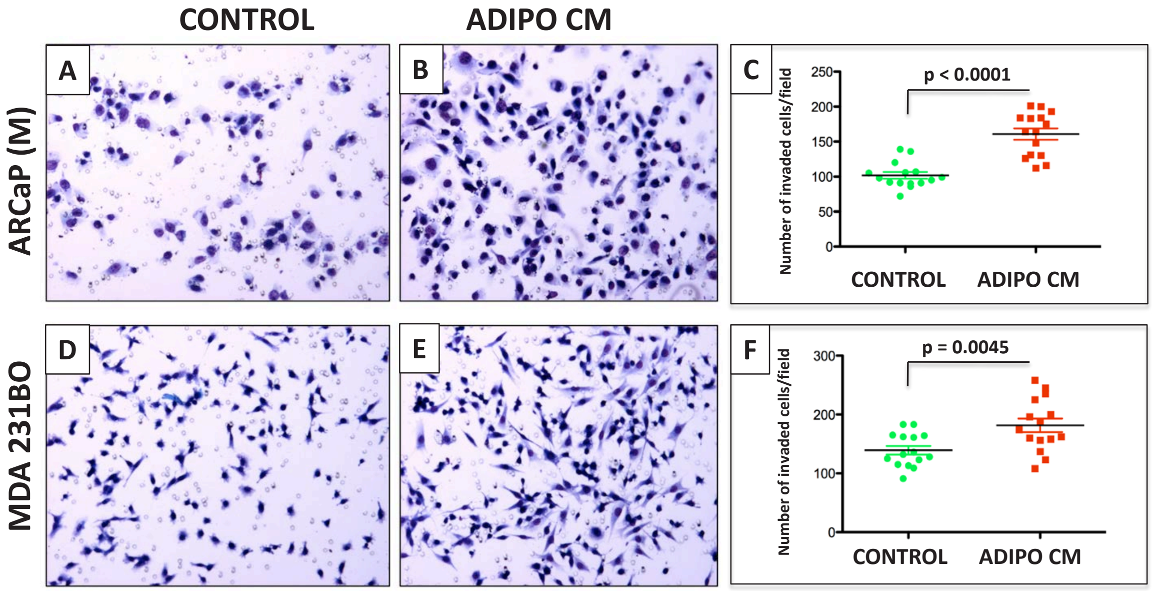

Supplementary Figure 8. Chronic exposure to low dose Adipo CM increases invasiveness of ARCaP(M) and MDA-231BO cells. Cells were exposed to gradually increasing concentration of Adipo CM (gradient of 5-25%) and maintained in 25% Adipo CM over multiple passages. Prior to assay cells were serum starved, seeded in serum free media on top of rBM-coated filter and allowed to invade towards DMEM with 10% FBS for 48 hours. Diff-Quik stained invasion filters for ARCaP(M) (A, B) and MDA 231BO (D, E) cells. C, F: Quantification results showing numbers of invaded cells.

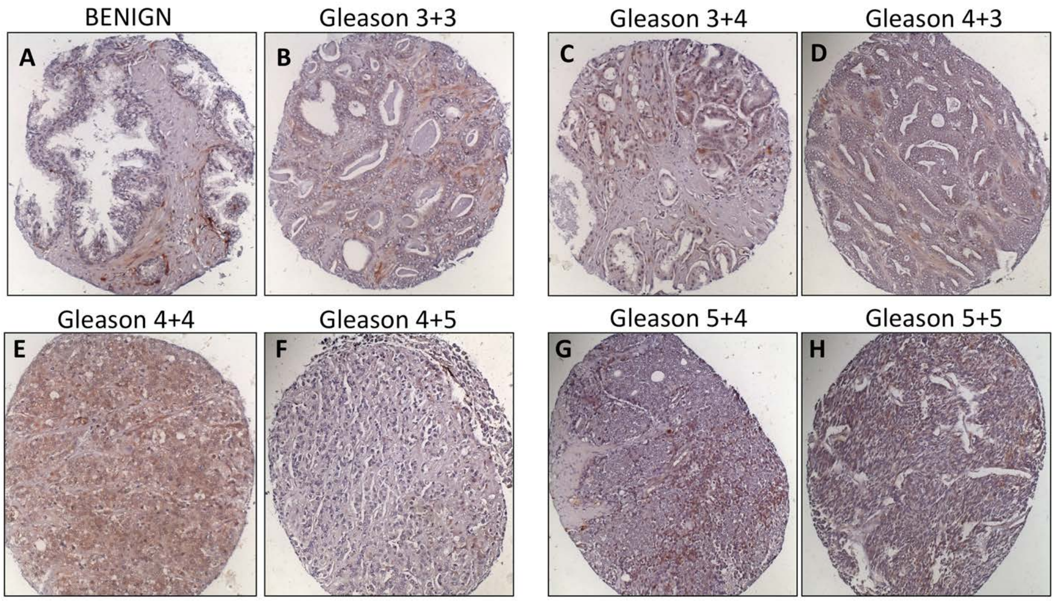

Supplementary Figure 9. FABP4 expression in normal and tumor tissue from prostate cancer patients. FABP4 immunostaining of TMA sections from normal prostate (A) and primary prostate tumors (B-H). B: Gleason 3+3; C: Gleason 3+4; D: Gleason 4+3; E: Gleason 4+4; F: Gleason 4+5; G: Gleason 5+4; H: Gleason 5+5.

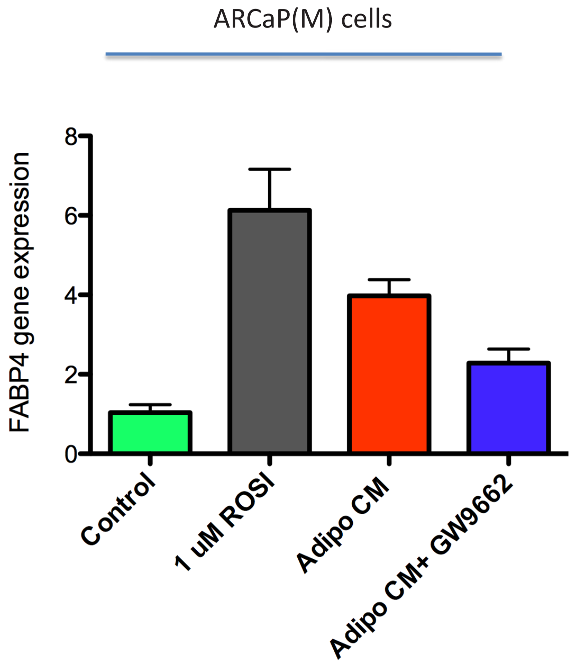

Supplementary Figure 10. FABP4 expression in ARCaP(M) cells is induced by Adipo CM and PPARγ ligand rosiglitazone. Taqman RT PCR analysis of FABP4 expression in ARCaP(M) cells grown in the absence or presence of 1μM rosiglitazone (ROSI), Adipo CM, and Adipo CM in the presence of PPARγ antagonist GW9662 (1μM). Data are normalized to 18S and shown as fold change relative to control cells. FABP4 expression increases with ROSI and Adipo CM and is reduced with GW9662.

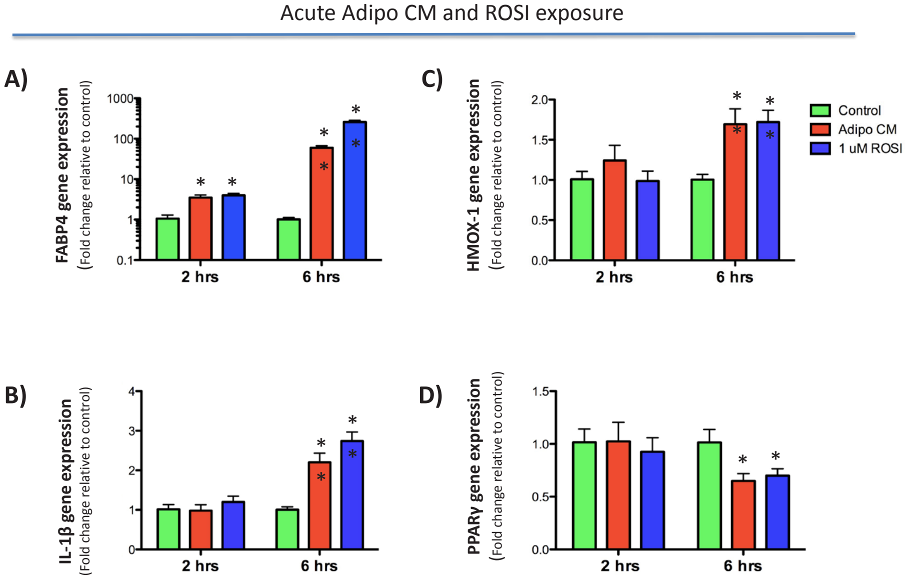

Supplementary Figure 11. Acute exposure to Adipo CM and Rosiglitazone modulates FABP4, IL-1β, HMOX-1 and PPARγ expression in PC3 cells. Taqman RT PCR analysis of FABP4 (A), IL-1β (B), HMOX-1 (C) and PPARγ (D) expression in PC3 cells cultured in the absence or presence of Adipo CM or 1μM rosiglitazone for 2 and 6 hours. Data are normalized to 18S and shown as fold increase relative to control. FABP4 expression is significantly increased at 2 hours of Adipo CM and ROSI treatment and increases in IL-1β and HMOX-1 expression are significant at 6 hours. PPARγ expression is reduced by Adipo CM and ROSI treatment after 6 hours. Values * $p < 0.05$; ** $p < 0.01$ are considered statistically significant.
